# Living with pulmonary fibrosis: how affected people experience disease-related information, health services and self-management strategies

**DOI:** 10.1136/bmjresp-2025-003303

**Published:** 2025-11-28

**Authors:** Thomas F Riegler, Thimo Marcin, Markus Wirz, Patrick Brun, Milo A Puhan, Sabina Guler, Anja Frei

**Affiliations:** 1 ZHAW Zurich University of Applied Sciences, Institute of Physiotherapy, Winterthur, Switzerland; 2 Center for Rehabilitation & Sports Medicine Inselspital and Berner Reha Zentrum, Bern University Hospital, University of Bern, Heiligenschwendi, Switzerland; 3 Department for Pulmonary Medicine, Allergology and Clinical Immunology, Inselspital, Bern University Hospital, University of Bern, Bern, Switzerland; 4 Institute of Epidemiology Biostatistics and Prevention, University of Zurich, Zurich, Switzerland

**Keywords:** Interstitial Fibrosis, Idiopathic Pulmonary Fibrosis

## Abstract

**Introduction:**

People with pulmonary fibrosis (PF) experience gaps in care, educational resources and self-management strategies throughout their journey. We sought to identify gaps in care and information, determine essential information, examine sources of information and assess preferred modes of delivery for patient education and self-management (PESM) resources and interventions.

**Methods:**

In this qualitative study, we conducted interviews with people with PF using predefined, literature-based categories (“staying well with PF”, “keeping fit & strong with PF”, “using oxygen therapy”, symptom management “breathlessness”, “cough”, “fatigue”, “anxiety, depression and panic”) and categories derived from own experience (“sources of information”, “preferred modes of education delivery”). Interviews were analysed using deductive-inductive content analysis.

**Results:**

We interviewed 11 individuals with PF (one woman), median age of 73 years (range 52–80) and time since diagnosis ranging from 1 to 10 years. Interviews lasted 40–70 min. A priori-defined saturation was reached for each predefined topic. Patients with PF relied on healthcare professionals (HCPs) as their primary information source. However, for sensitive topics such as life expectancy, death or when time with HCPs was limited, they often turned to internet searches with little success. There was a need for guidance on managing antifibrotic side effects, including adaptation of nutrition. While exercise was valued, it lacked structure, particularly at home. Self-management strategies for cough and fatigue remained insufficient. Digital resources were considered beneficial but maintaining in-person interactions with HCPs is essential.

**Conclusion:**

This in-depth analysis highlights how people with PF comprehend patient education content and which taught or self-acquired self-management techniques they implement in their lives, including beliefs and concerns. These insights are essential for refining current and future PESM programmes and support the creation of blended learning and digital resources, with HCPs offering guidance on their use, to further support people with PF in self-management.

**WHAT IS ALREADY KNOWN ON THIS TOPIC**

- People with pulmonary fibrosis (PF) experience gaps in care, educational resources and self-management strategies throughout their journey.

**WHAT THIS STUDY ADDS**

- To our knowledge, this is the first study to conduct an in-depth analysis of how people with PF comprehend patient education content and which taught or self-acquired self-management techniques they implement in their daily lives. From the perspective of people with PF, this study expands on the understanding of patient education, the real-world application of self-management strategies and sources of information, while also exploring future digital service modes in patients with PF. We found gaps in symptom management for cough and fatigue or sensitive topics such as life expectancy, death or sexuality. We also address opportunities for blended learning.

**HOW THIS STUDY MIGHT AFFECT RESEARCH, PRACTICE OR POLICY**

- This study provides researchers, healthcare professionals and educators with valuable insights into the experiences and beliefs of people with PF, informing the development and improvement of patient education resources and guiding coaching sessions.

## Introduction

 Pulmonary fibrosis (PF) is a life-altering disease characterised by decline in exercise capacity and quality of life.^1^ People affected by PF suffer from breathlessness, cough, fatigue, anxiety/depression and face treatment-associated as well as end-of-life challenges.^2^,^6^

Patient education and self-management (PESM) information, strategies and techniques are essential for empowering patients in the day-to-day disease management and in pulmonary rehabilitation.^7 8^ There is a need to enhance self-management support for people with PF, especially to provide adequate information and strategies to relieve symptoms.^36 9^,^12^ Internet resources are often incomplete and sometimes inaccurate.^13^,^16^ Self-management packages have been suggested,^17 18^ some PESM resources have been co-written by people with PF, carers and clinicians^19^; however, facilitators and barriers to adoption still need to be demonstrated.

We aimed to explore experiences of people with PF regarding patient education resources and self-management techniques developed based on pre-established domains.^4 5^ Additionally, we sought to identify specific gaps in care and information and determine the most helpful and comprehensive information as perceived by people with PF. Finally, preferred sources and modes of information delivery for educational and self-management resources and interventions were explored, with the goal to inform and refine existing and future PESM programmes for people with PF.

## Methods

### Study design

We conducted an exploratory qualitative study using interviews with people affected by PF.^20^ Data collection was part of a multiphase mixed-method study to establish consensus on PESM content in people affected by PF and healthcare professionals (HCPs), which ultimately provided the basis for the development of a PESM programme. The Standards for Reporting Qualitative Research were used.^21^

### Patient and public involvement

Patients and members of the public were not involved in the design, conduct, reporting or evaluation of this study. Patients contributed as research participants. The research question and outcome measures were informed by patient experiences and preferences reported in previous studies. The burden of participation was not assessed. The results of this study will be incorporated into a blended PESM programme and disseminated in collaboration with the Swiss patient association for PF and the Swiss Lung Association.

### Theoretical framework for semi-structured interview guide

We developed a semi-structured interview guide using predefined, literature-based categories as a theoretical framework (online supplemental file 1).^22^ In short, we used the core education topics and subtopics as previously suggested as categories, including “staying well with PF”, “keeping fit & strong with PF”, “using oxygen therapy”, “symptom management of ‘breathlessness’, ‘cough’, ‘fatigue’ and ‘anxiety, depression and panic’”.^4^ We supplemented these with subtopics, which were selected based on consensus results and redundancy, such as “accessing community support”, “understanding treatment options for PF”, “managing medications—including side effects, role and importance of pulmonary rehabilitation”, “advance care planning and advance directives”, “managing comorbid medical conditions” and “recognising an exacerbation”.^5^ Furthermore, we added self-identified questions about “resources of information”, “use of support services” and “delivery modes of PESM contents”. We based our decision to include/exclude topics for the interview guide on our experience of their relevance to the daily lives of people with PF.

### Participants

Between December 2022 and March 2023, people with PF who presented at the outpatient PF clinic of the University Hospital of Bern, Inselspital, or the inpatient pulmonary rehabilitation clinic Berner Reha Zentrum AG, Switzerland, were presented with flyers about the project. Patients contacted us on their own initiative to share their experience with PESM material. A priori, the required sample size for patient interviews was determined when data saturation of the predefined categories was reached.^20^

### Data collection and analysis

All interviews were conducted in German and online via Webex, recorded using TechSmith Camtasia, V.2022.5.2 and exported to MP3 by the same researcher (TFR), a physiotherapist with 8 years’ experience in pulmonary rehabilitation in PF. Interviewees guided the direction of the narration of their experiences, while the interviewer specified the context or deepened on topics based on the semi-structured interview guide.^20^ If interviewed persons reported a subtopic not covered by the deductively derived categories, a new category was established inductively.

Interviews were transcribed verbatim and analysed in a content structuring approach, a subcategory of content analysis based on Mayring (p. 103).^23^

Analyses were performed assuming a constructivist worldview.^24^ We performed an investigator triangulation to enhance consistency and credibility.^25^ Each interview was coded independently by two investigators, and all interviews were subsequently analysed by another investigator (three investigators in total). A coding guide led the coding process (pp. 111–113).^23^ Additionally, the primary coder (TFR) instructed both other coders to increase reliability of the process.

All identified codes were collapsed into broader themes inside the deductively predefined or inductively newly generated categories to establish the final code tree using MAXQDA software (V.2022).^26^ The content per category was presented as narrations accompanied by representative quotes based on the emergent code tree. The narration structure was developed through discussions among all three coders to determine the most suitable interpretation of the data.

## Results

Participants had a diverse mix of educational and professional backgrounds and felt “sure” to “very sure” in managing their disease in daily life (table 1). In total, 10 hours of interviews were performed, each lasting from 40 to 70 min. The predefined categories covered all reported topics and were subsequently filled with narrations based on the generated code-tree. The categories are presented narratively, including direct quotes from the participants. Additional representative quotes for each category are provided in online supplemental table S1 to offer further insights.

**Table 1:** Participant characteristics

Total, n (%)	11 (100%)
Women, n (%)	1 (9%)
Age in years, median (range)	73 (52–80)
Educational backgrounds	Vocational training, n=7 Advanced vocational school, n=1 Higher education, n=3
Recruited from, n (%)	
University Hospital of Bern	5 (45%)
Berner Reha Zentrum AG	6 (55%)
Diagnoses, n (%)	
Idiopathic pulmonary fibrosis	5 (45%)
Unclassifiable pulmonary fibrosis	5 (45%)
Progressive fibrotic hypersensitivity pneumonitis	1 (10%)
Lung transplantation, n (%)	2 (18%) 2 and 6 years ago, respectively

**Figure 1:**
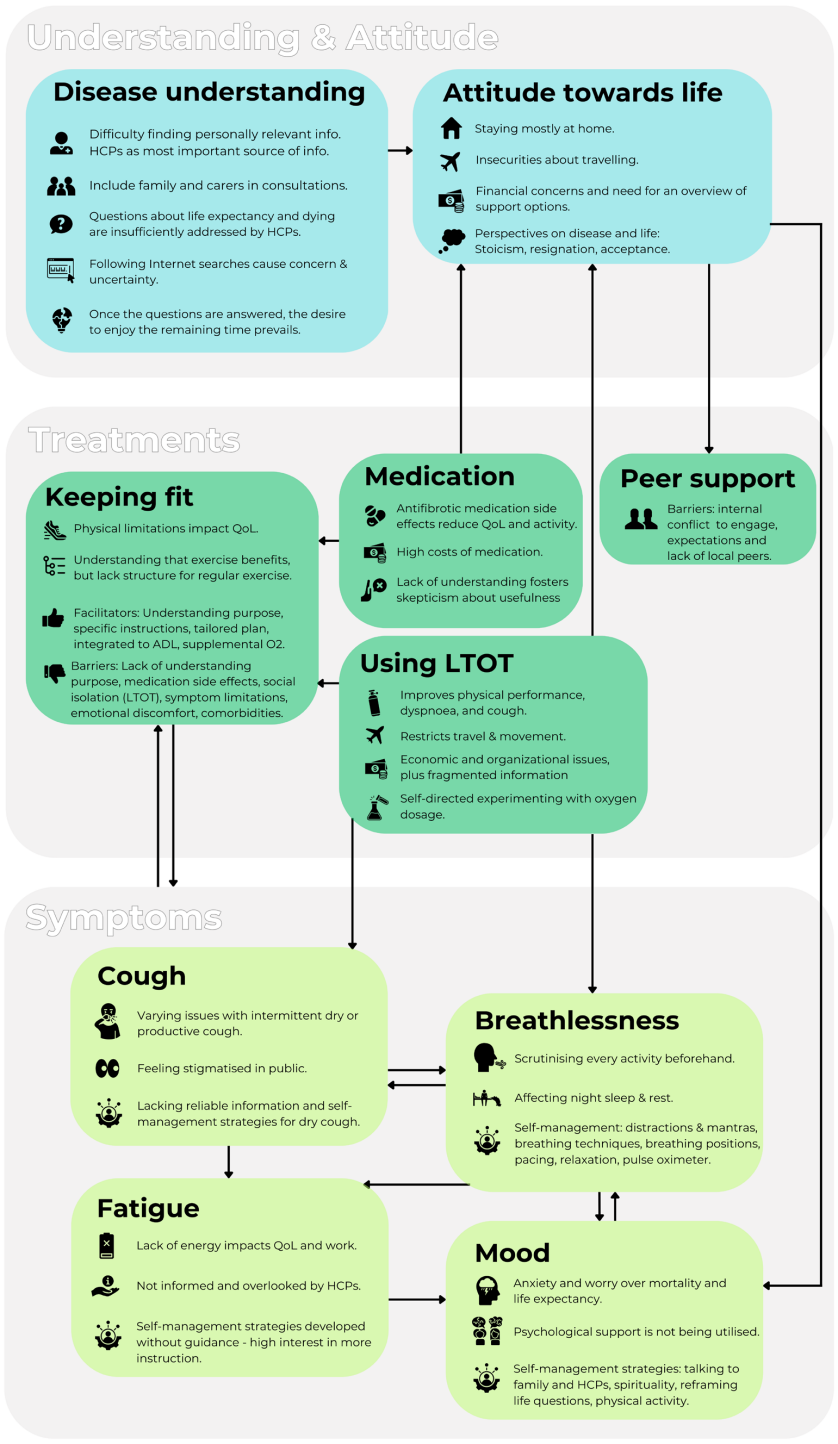
Interrelationships between key experiences within literature-based categories, as reported by people living with pulmonary fibrosis (PF). Each category encompasses the most significant factors identified, and the arrows indicate the connections between them as experienced by people with PF based on the interview data. ADL, activities of daily living; HCP, healthcare professional. LTOT, long-term oxygen therapy; O_2_, oxygen; QoL, quality of life.

The interrelationships between key experiences as reported by people with PF are shown in figure 1.

### Staying well with PF

#### Understanding the disease

All interviewees reported that their HCPs were the most important source of information. Inclusion of family members in communications about PF seemed important. The interviewees knew that there are many forms of PF and acknowledged that this made finding reliable and personally relevant information more challenging. Some interviewees stated that they knew little about their disease and were not able to explain it to others. The remaining found the following expressions useful: PF is an “incurable” lung disease with most often “unknown origin (idiopathic)”, where “scarring” occurs, which leads to the lungs becoming “rigid” and the “alveoli harden”, and ultimately “preventing effective gas exchange”. The lungs then look “like a spiderweb”, they become “compact”, resulting in a “reduced lung capacity, which also means reduced breathing capacity”. The “lung deterioration leads to low oxygen levels, affecting all organs” and “there may be progressive deterioration or exacerbations”.

Interviewees stated that they were aware of the importance of infection prevention by getting all recommended vaccinations, wearing facemasks during flu season and a healthy lifestyle with physical activity and thoughtful nutrition.

#### Life expectancy

For most patients, the immediate and pressing questions following diagnosis were, “How long will I live?” and “How will I die?” However, these questions were not always addressed by HCPs, or not in sufficient detail. Consequently, patients turned to the internet for answers and/or became preoccupied with these thoughts. Most had come across survival estimates online suggesting a lifespan of 2–5 years and were left to process this information on their own. In particular, the fear of “suffocating due to breathlessness” was deeply distressing. Learning that morphine could make the dying process more comfortable helped to alleviate this concern for many participants.

Patients knew that antifibrotic medications can extend life expectancy and that lung transplantation remains the best option for long-term survival. For the majority, end-of-life planning was considered important. A checklist to facilitate discussions with HCPs and family members was perceived as desirable. Completing an advance directive, preparing a will, discussing wishes for emergency situations with family and organising personal and financial matters were mentioned as important. In general, patients expressed a desire to communicate openly with HCPs about their life expectancy.

#### Attitude towards life with PF

PF restricts the lives of those affected, and, as a result, some no longer leave their homes. Some reported that they wished to travel and more information on “how and under what conditions this could be achieved”.

The helplessness of suffering from an incurable disease burdens their thoughts. In some cases, people with PF seemed to project their own despair onto the healthcare providers “they don’t really know what to do either”. Those with idiopathic PF often had a strong desire to understand the cause as to “why do I have this?” Some patients felt overwhelmed by the disease, stating that “I never thought it would progress so quickly” and, due to the burden of symptoms, became irritable “then I screamed to everyone: ‘of course I’m greatly irritated!’”.

Financial concerns were reported and expressed as the need for an overview of available support (such as pension compensation, disability benefits and financial support). Additionally, patients wanted to advocate for themselves within the healthcare system and hoped for greater empathy from HCPs, especially their physicians. However, they did not want pity.

Participants acknowledged that PF requires lifestyle adaptations, for example, regular exercising, or they felt PF was something they “must just endure.” Generally, a sense of acceptance was reported due to age “I’ve had a good life” or acceptance of the inevitability of dying “you have to die sooner or later”, religion or spirituality, fate and positive thinking “compared to others, I’m doing well”. Only one patient appeared resigned and preferred not to think about PF.

#### Medication

Medication is a central aspect for patients and occupies a significant space in their daily lives. Patients were critical of their long lists of medications as they felt that some had little effects or even undesired side effects. Particularly, the impact of antifibrotic medication side effects on quality of life was highlighted, “I’m constantly dealing with side effects, and then additional medications for those side effects.” Some patients reported that the purpose of the medication was often insufficiently explained, while the list of side effects was overwhelming and difficult to process. This led to doubts about the usefulness of the medication. Patients mentioned that HCPs are overly focused on the medication. “I tell them about the side effects, and how much they limit my life, and the response is, it doesn't matter, I still have to take it.” Side effects of antifibrotic medications were mentioned most frequently and included diarrhoea, loss of appetite or nausea, with a significant reduction in quality of life.

Morphine was positively mentioned by some patients as a means of “not having to suffer a terrible death by suffocation”

#### Nutrition

Nutrition was considered an important aspect, with many patients experiencing medication side effects. Generic dietary advice was perceived as unhelpful. Most valuable were recommendations on how nutrition can help alleviate medication side effects.

#### Peer support

Support from other patients was perceived in mixed ways. A key benefit was to learn from shared experiences. Discussing the disease seemed helpful and made some feel less alone. Others expressed no interest in peer connection, “I have absolutely no interest in celebrating my disease with others”, noting that different people have different goals. A few patients felt pressured to offer advice to others. Barriers to peer support included an internal conflict about engaging in peer interactions and the lack of peers in their area.

### Keeping fit and strong with PF

All interviewees acknowledged that their limitations to physical capacity impacted most areas of their life. Despite understanding that physical activity and exercise are important, most reported generalised statements: “Basic fitness is important for everyone” and “Movement is good for the lungs and muscles”. Patients reported a lack of detailed explanations of “why one should stay active, even though it’s difficult due to breathlessness” as a **barrier** to physical activity and training. Additional barriers included medication side effects, social isolation (eg, due to oxygen therapy), symptom-related limitations, breathlessness and fatigue, poor psychological or emotional well-being, overcoming lack of willpower and comorbidities (eg, orthopaedic issues).

Family members acted as either barriers or facilitators to physical activity. Similarly, weather conditions and the mode of training, individual vs group settings, or the appeal of the training played a role.

Facilitators included understanding the purpose and benefits of physical activity, practical recommendations “exercise x times per week!”, individually tailored home training plans, a positive attitude towards exercise and a “push-through” mentality. “Feeling better afterwards” served as a motivator. Supplemental oxygen facilitated activity by improving breathlessness. Combining exercise with daily activities “I do my cycling in front of the TV” was also helpful. Pulmonary rehabilitation was reported as a strong motivator for continuing training at home.

Home training was generally acknowledged as a “necessary task” and often carried out unstructured and largely without specified goals. Exercise programmes were frequently mentioned and included exercises for strength, endurance and breathing. Most endurance training was described as walking, (stationary) biking and stair climbing, mostly without a defined training stimulus. Occasionally, training was incorporated into daily activities, “I’m taking the stairs instead of the elevator.” Notably, some patients made use of training programmes designed for chronic obstructive pulmonary disease (COPD).

### Using oxygen therapy

Patients reported that oxygen therapy was essential and related certain symptoms to low oxygen levels, for example, difficulty finding words, forgetfulness, headaches, cold or bluish extremities, feeling of the body “blocking”, reduced physical performance or waking at night with breathlessness. Some patients self-monitored with a pulse oximeter.

#### Positive and negative impacts of oxygen therapy

Interviewees stated that oxygen therapy improved their physical performance by alleviating breathlessness and reducing cough. Negative aspects included avoidance of travel, the need for a cumbersome backpack and feeling tethered to oxygen devices. Overall, freedom of movement and autonomy were tightly linked to the availability of refill stations for liquid oxygen devices. Patients also complained that the choice of oxygen device and type is influenced by economic and local factors, for example, inability of delivery, rather than personal preference.

#### Information

Information on oxygen therapy should include charts for comparing oxygen devices, driving, travelling and different application methods like oxygen cannula, masks and oxygen reservoir cannula, including possible abrasions on, for example, ears. Patients received information from the Lung Association, gas suppliers and brochures which some perceived as too fragmented.

#### Experimenting with oxygen dosage

Patients reported self-directed experimenting with oxygen dosage. Some monitored their oxygen level and appropriately adjusted their dosage during physical activity. However, less structured experiments were also reported, such as reducing oxygen intake to “make the lungs work harder”, using unstructured “convenience/whim” or adjusting oxygen according to “their feeling”. Some occasionally tested to sleep without oxygen and woke up breathless.

#### Travel

This required meticulous organisation. Some people with PF planned the duration of journeys according to the available oxygen supply and refill stations and conducted strategic use of oxygen dosage to extend autonomy. Travel abroad involved power supplies, battery issues, higher oxygen requirement during air travel and restrictions of specific devices on specific airlines. Most interviewees stated they felt limited in their travelling abilities and that using a car was the only solution for them. Few patients added that they used bigger cars to accommodate 20 L liquid oxygen tanks during travel to enhance mobility.

### Managing symptoms

#### Breathlessness

All interviewees reported breathlessness as the primary symptom, predominantly during physical activity, leading to scrutinising activities beforehand, “You constantly ask yourself, ‘Do I really have to do this now?’”, limiting participation, “I can no longer visit my friends the way I used to.” Breathlessness also affected sleep.

Although patients considered information on managing breathlessness as important, most knew only unstructured and limited self-management techniques. They reported strong interest in “How can I quickly return to a normal breathing rhythm?”, “How can I adjust my activities to minimise breathlessness?” and “Provide many relief positions so I can choose the one that works best for me.”

Core elements for short-term dyspnoea management included cognitive strategies, like redirecting thoughts, breathing techniques, for example, post-inspiratory pauses, and body positions, for example, lying with supported arms, retreating to a quiet place, fresh-air ventilation and morphine. These techniques prevented a feeling of panic, “Because I knew it would pass if I waited long enough, I wasn’t actually afraid of it anymore.”

Long-term management of breathlessness included physical exercises, adjusting activity intensity along with respiratory pacing, relaxation and delegating strenuous tasks. Patients also valued using a pulse oximeter.

#### Cough

There was variability regarding dry and productive coughing ranging from rare to very frequent and from a minor issue to life-limiting. Stigmatisation was reported, especially since the COVID-19 pandemic, “I get stared at harshly when I have to cough on the bus.”

Dry cough was perceived as the most disruptive and increased fatigue. Patients reported that intense respiration triggered dry cough. Other triggers included exercise, cold/dry air, powders and low oxygen saturation. The uncertainty about the cause of dry cough preoccupied patients. Most tried cough self-management techniques, while only a few reported that they simply “coughed until it stopped”. Some mentioned self-medication with codeine-based cough syrups, with little success. Strength and breathing exercises were described as initially cough triggering with relief when continuing exercise. Reported cough suppression techniques had inconsistent benefit (eg, elevating the upper body in bed, throat clearing or swallowing, adjusting breathing depth, drinking water or sucking on lozenges).

#### Fatigue

All interviewees reported fatigue and feeling “tired”, “exhausted” or “worn out”, with their body feeling “heavy” or like it is made of “lead”. Overexertion led to “dizziness” and “balance issues”. Symptoms fluctuated during the day and were associated with activities or dry cough.

Patients stated they had not learnt techniques for dealing with fatigue during consultations or rehabilitation. Daily activities were spread throughout the day. “Pacing” included performing tasks more slowly or with frequent breaks, for example, lying down or sleeping. Workdays needed to be adapted with employers, reducing time at work or adding breaks.

Many mentioned that carefully dosed activities reduced fatigue. Yet, they expressed an ambivalence about being less active despite knowing the benefits. Overexertion through activities was mentioned as counterproductive with extended recovery times, emphasising careful dosing of activities.

#### Symptoms of anxiety, depression and panic

Psychological symptoms were primarily mentioned in the context of dying. The uncertainty about life expectancy was a significant burden. Some reported feeling torn: “Why keep fighting, but at the same time, I want to live a little longer.” Negative thoughts arose from the helplessness of an incurable disease and led to unrest or depression. Some individuals avoided engagement with their illness, as it felt too overwhelming, “[…] but somehow, I don’t really want to know more about it … it pulls you into a hole.” Others did not want to burden their friends or family with their mental state. Some reported that psychological stress also led to physical inactivity.

Family support was a crucial coping strategy and a space for discussing these thoughts. Validating and allowing emotions helped individuals to cope more effectively. Most were offered psychological support; however, only a few took advantage of this. Some relied on religion or spirituality for support, while others practised “reframing” by categorising their thoughts as the larger questions of life, thus normalising them. Some interviewees mentioned that physical activity significantly lifted their mood.

### Sources of information

Patients primarily relied on HCPs and the internet, along with information brochures and family or friends. They placed importance on being treated by a specialised medical doctor for PF and emphasised the wish for opportunities and time to ask questions. Easily accessible, informal phone contact with the PF centre was also highlighted as a resource. Pulmonary rehabilitation and the regional Lung Associations were reported as a source for self-management and knowledge. Family members were often involved in information gathering. Searches on the internet were used by all interviewees, but mostly with limited success. Searches were viewed as time-consuming and in the end, “the information couldn't really be trusted and made me insecure about everything”. The internet was primarily used for sensitive topics which were not addressed by HCPs, especially regarding life expectancy, death and sexuality.

### Modes of information delivery

Most interviewees reported that they preferred a blended learning approach, that is, mixed digital and face-to-face sessions. Digital information sources were seen as beneficial for self-directed, interest-based learning as opposed to group sessions in pulmonary rehabilitation. All interviewees reported that they would be able to access a digital resource and, if necessary, would be supported by others. Some interviewees noted that few people might have trouble in accessing digital resources and recommended paper-based backups.

All interviewees stated that face-to-face sessions with HCPs either in an individual or group setting were essential for discussing the information and techniques learnt from a digital programme.

Further topics were preparing for medical consultations, lung transplantation and accessing support services from institutions.

**Figure 2:**
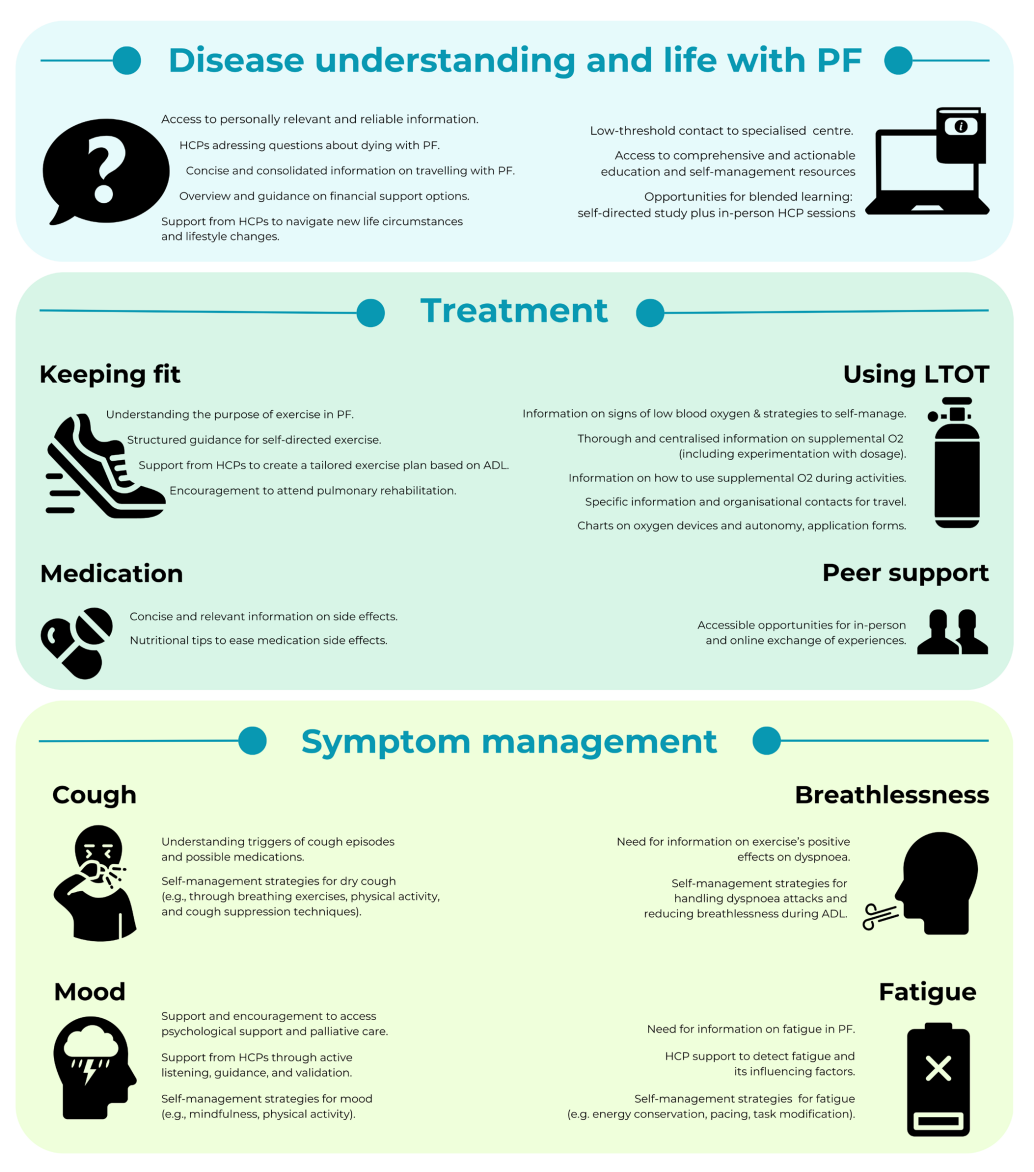
Overview of gaps and needs in patient education and self-management within literature-based categories, as reported by people living with pulmonary fibrosis (PF). ADL, activities of daily living; HCP, healthcare professional; LTOT, long-term oxygen therapy; O_2_, oxygen.

Figure 2 provides an overview of the gaps and needs in patient education and self-management, as reported by people with PF.

## Discussion

This qualitative study explored experiences of individuals with PF regarding self-management strategies and identified specific gaps in care and information. Additionally, participants shared their preferences for the format and delivery of educational and self-management resources. To our knowledge, this is the first study to conduct an in-depth analysis of how people with PF comprehend patient education content and which taught or self-acquired self-management techniques they implement in their daily lives. These insights are essential for refining current and future PESM programmes.

For all interviewees, the primary source of information was their healthcare team, which was limited by the physicians’ availability and the scope of services provided. Internet-based searches emerged as the second most used source of information, despite the inherent uncertainty of its accuracy and credibility. Online searches were adopted for questions individuals were unable or unwilling to address with their healthcare team due to fear, shame or stigma. Previous studies have reported that online PF information is often inaccurate, incomplete, outdated, misleading or difficult to find.^13^,^16^ This aligns with our findings and underscores the need for reliable sources of (online) information, dedicated time for HCPs and effective “icebreakers” to address sensitive topics often linked to stigma, shame or fear (eg, sexuality, life expectancy or death). Furthermore, our findings align with and confirm the educational needs and priorities identified in a previous study, while extending this work by exploring patients’ experiences with these needs.^27^

Unlike in other studies,^6 11 28^ our interviewees acknowledged the benefits of peer support but were reluctant to participate in peer support groups. This may be attributed to cultural differences or reflect a lack of suitable support groups, likely due to the rarity of the disease and physical limitations limiting travel with PF. Online video meetings might effectively address this gap.

Pulmonary rehabilitation and ongoing coaching with an exercise plan are key components for patients with chronic respiratory diseases.^8^ Our data highlight the need to support patients to remain active outside of structured settings such as pulmonary rehabilitation.

Despite cough and fatigue being common and impactful,^29 30^ our data indicate that patients employ few self-management strategies. Fatigue appears to be frequently overlooked by HCPs. While patients in our sample intuitively adopted strategies to manage fatigue, there remains a need to enhance self-management coaching for both fatigue and cough. Overall, our interviewees demonstrated very active engagement in symptom management, emphasising the crucial role of these techniques in daily life.

Our interviewees appreciated the concept of blended learning, including self-directed learning. Blended learning has been shown to be effective in improving self-management practices in COPD,^31^ is a highly effective method for delivering medical content to healthy subjects^32^ and can promote learning autonomy.^33^ Few interviewees emphasised the need for paper-based educational resources, but recent studies have demonstrated that digital resources are a feasible means of delivering self-management education in PF.^17 34^ Therefore, we conclude that the exploration of digital resources for PESM is appropriate.^35^ These modern modes of education might help overcome barriers to group sessions.^6^ Future blended PESM programmes for PF could incorporate tailoring functions to help guide patients’ use of comprehensive digital resources that provide actionable self-management techniques. By using a tailored blended learning approach, people with PF could prepare before in-person or digital meetings with HCPs to refine their self-management plans and address their specific questions.

A limitation of this study is the high proportion of men with PF, which resulted from the consecutive recruitment process. Although PF is more prevalent in men, this ratio may not accurately reflect the general PF population,^36^ potentially leading to a gender bias. Furthermore, our participants represent a sample of well-informed people with lived experience, recruited from specialised centres. This was suitable to answer our research question, but may not reflect the population of people with PF.

Interviews are considered more suitable for collecting in-depth personal experiences.^20^ However, focus groups could have revealed additional topics. We interviewed only 11 people with lived experience. While there are no fixed rules for sample sizes in qualitative research, a rule of thumb suggested that 10–20 interviews are sufficient to understand established categories when studying lived experience.^37^ Using literature-based theoretical categories, we were able to reach data saturation for both core topics and subtopics relatively quickly. Therefore, we are confident that the data collected is representative for our study’s purposes. However, with only 11 participants, we were unable to identify potential differences in experiences between IPF and other forms of PF, or between pre-lung and post-lung transplantation.

In conclusion, this study highlights specific areas, beliefs and concerns of patients with PF that can inform the development of patient education resources and guide HCPs. In addition to research evidence and expert clinical experience, it is essential that self-management techniques used by people with PF are included into existing or future educational materials, emphasising the inclusion of people with lived experience as active partners in research and innovation to improve their care. Lastly, we emphasise the importance of HCPs referring patients to reliable online patient education resources.

## Supplementary material

10.1136/bmjresp-2025-003303 online supplemental file 1

10.1136/bmjresp-2025-003303 online supplemental file 2

## Data Availability

Data are available upon reasonable request.
