# Supplementary material for: Living with pulmonary fibrosis: how affected people experience disease-related information, health services and self-management strategies
Source: BMJ Open Respir Res. 2025 Nov 28;12(1):e003303. doi: 10.1136/bmjresp-2025-003303 (PMC12666047; doi:10.1136/bmjresp-2025-003303)
Supplement: online supplemental file 1 [file bmjresp-12-1-s001.docx]

**SUPPLEMENT 1 – Interview Guide**

**Living with pulmonary fibrosis: How affected people experience disease-related information, health services and self-management strategies**

Thomas F. Riegler^1^, Thimo Marcin^2^, Markus Wirz^1^, Patrick Brun^2,3^, Milo A. Puhan^4^, Sabina A. Guler^3^ and Anja Frei^4^

**Affiliations:**

^1^ ZHAW Zurich University of Applied Sciences, School of Health Sciences, Institute of Physiotherapy, Winterthur, Switzerland

^2^ Berner Reha Zentrum, Rehabilitation and Sports Medicine, Insel Group, Bern University Hospital, University of Bern, Bern, Switzerland

^3^ Department for Pulmonary Medicine, Allergology and Clinical Immunology, Inselspital, Bern University Hospital, University of Bern - Bern (Switzerland).

^4^ Epidemiology, Biostatistics and Prevention Institute, University of Zurich, Switzerland

**Correspondence:**

Thomas F. Riegler

ZHAW Zurich University of Applied Sciences, School of Health Sciences, Institute of Physiotherapy

Katharina-Sulzer-Platz 9

8401 Winterthur

Switzerland

E-Mail: thomas.riegler@zhaw.ch

# Semi-structured interview guide

** This interview guide was translated automatically from German.*

**Objective(s) of the interviews:**

**Overall goal**

- The results of these interviews will be used to create a comprehensive list of contents for self-management and training of patients with pulmonary fibrosis. This list of contents includes experiences, tips on how to manage symptoms, and exercises that are important for pulmonary fibrosis patients.

**Sub-goals**

- Patients can share their experiences with self-management techniques, exercises and important information in an unbiased way in order to generate a list of important self-management and training content based on these experiences.
- It should also be possible to place wishes for additional information / self-management techniques that they consider particularly important in order to be able to expand the list of contents more comprehensively, beyond their own patient experiences.
- Patients should be able to "save" particularly valuable information or self-management techniques/exercises/tips so that they cannot be removed from the list of contents in the Delphi expert rounds. This is intended to strengthen patient focus.

**Procedure/structure of the interviews**

- Open beginning and end to allow patients to communicate easily, intuitively.
- In the middle part of the interview, unaddressed areas of the 'Core Education Topics' (see below in **bold**) will be systematically queried. This is intended to ensure completeness.

**Welcome, Introduction**

**Information at the beginning**

- **Why** – get to know the experiences and opinions of people with pulmonary fibrosis in order to incorporate them into the creation of a self-management program (those affected as experts for their disease)
- **What do we want to learn from this** – valuable information / exercises / tips / handling for those affected, which they apply in order to be able to deal better with their illness/symptoms and to keep fit for as long as possible,
- **What are results used for –** based on these interviews, content lists with important training information, tips and self-management techniques are created, which are then specified/selected in further expert panels.
- **Procedure** – beginning with some open questions about experiences – if indicated, specific symptoms are still asked.
- Info: Questions may also remain unanswered. Things/info/tips that are particularly helpful can be specially 'marked'.
- Duration 30-60 minutes

**Assessment of own self-management skill:**

- From 0-3; How safe do you feel in dealing with your illness in everyday life?
- 0 – I feel very insecure about how to deal with my illness in everyday life.
- 1 – I feel rather insecure about how to deal with my illness in everyday life.
- 2 – I feel more confident about how to deal with my illness in everyday life.
- 3 – I feel very confident in how to deal with my illness in everyday life.

**Start with open questions:**

- What **information** has helped you to **understand your illness** and which of it seems valuable to you? What **information** has helped you **to better understand your symptoms of illness**? Or what information has helped you to **positively influence your disease symptoms**?
- What **exercises or techniques** help you to  **positively influence** your disease symptoms?
- How do  **you communicate** to others what it's like to live with your illness? Who do you talk to about your disease? How were their caregivers involved in the process of their treatment? What would you have wished for?

**Specific questions on the core education topics and subtopics:**

- Have you ever been to pulmonary rehabilitation? If so; When approx.? Inpatient or outpatient?
  - Did you learn anything there about disease management or dealing with your own illness? Have you received valuable information for yourself?
- What general recommendations did you receive? Thought support for categories that could/should be included (will be asked if unclear, or something is missing): Vaccinations / Importance of training / Nutrition tips / Dealing with infections – deterioration? If so, what?
  - Which of these tips were particularly helpful for you? Which ones not at all?
  - Which substances or situations cause you particular trouble in everyday life?
- Are they **physically active**, or do they exercise (e.g. fitness, housekeeping, gardening, walking, etc. – generally activities that make you sweat or sniff)? If so, how regularly/frequently?
  - What helped you to stay fit and active after rehabilitation?
    - If not; What would help you to take up physical activity?
  - What do you do to stay fit? What information/recommendations have helped you to stay fit?
  - What would you think is important for all people with this condition to know about training and its effects?
  - What helps you to motivate yourself?
  - Which breathing technique helps you the most during training?
- Do you have experience with supplemental **oxygen?**
  - What valuable information did you receive on this? What should every person with your disease know?
- Do you have or have you had **shortness of breath** and have you been informed about how to deal with it?
  - What information has helped you to better understand shortness of breath?
  - What do you think are the best ways to manage shortness of breath?
- Would it be important for you to learn information about **coughing** in particular in your illness? What should every person with your condition know about coughing? What understanding of the mechanisms should be conveyed?
- Do you have or have you sometimes had pronounced **fatigue** or poor performance? How do you deal with this? Have you received any recommendations that were particularly helpful in this regard?
- **Anxiety, depression and/or panic**: Are these topics on your mind? Have you received recommendations on how to deal with it? What are the most important tips?
  Do you have any tips on how to **manage emotional fluctuations** caused by the illness situation? What helps them?
- Which **providers** of help or services for the home do you find particularly important? Examples would be Spitex, meals on wheels, and much more. Which of them should all people with your disease be aware of?
- What tips for **traveling** with your illness do you think are important?
- What preparations do you make for a medical consultation?
- Is **end-of-life planning** a topic that you have dealt with because of your illness? If so, what information helped you? Which ones were you missing? What should patients know about this? Who would you like to receive it from?
  - *If no (be careful!);* was the topic addressed by their treating physicians/therapists?
- What information about your **medications** (as well as side effects) did you receive that seemed particularly important to you? Are there any topics that have particularly helped you in the past, or what would interest you?
- Do you have any **other illnesses**? If so, which ones? Would you have liked to receive information on these diseases as well?
- Which **training mode** could you imagine to be suitable (e.g. frontal/physical, digital)?
- Is there anything else that we haven't addressed yet that would be particularly important for patient education or self-management?
