# Supplementary material for: Living with pulmonary fibrosis: how affected people experience disease-related information, health services and self-management strategies
Source: BMJ Open Respir Res. 2025 Nov 28;12(1):e003303. doi: 10.1136/bmjresp-2025-003303 (PMC12666047; doi:10.1136/bmjresp-2025-003303)
Supplement: online supplemental file 2 [file bmjresp-12-1-s002.docx]

**SUPPLEMENT 2 – Quotes**

**Living with pulmonary fibrosis: How affected people experience disease-related information, health services and self-management strategies**

Thomas F. Riegler^1^, Thimo Marcin^2^, Markus Wirz^1^, Patrick Brun^2,3^, Milo A. Puhan^4^, Sabina A. Guler^3^ and Anja Frei^4^

**Affiliations:**

^1^ ZHAW Zurich University of Applied Sciences, School of Health Sciences, Institute of Physiotherapy, Winterthur, Switzerland

^2^ Berner Reha Zentrum, Rehabilitation and Sports Medicine, Insel Group, Bern University Hospital, University of Bern, Bern, Switzerland

^3^ Department for Pulmonary Medicine, Allergology and Clinical Immunology, Inselspital, Bern University Hospital, University of Bern - Bern (Switzerland).

^4^ Epidemiology, Biostatistics and Prevention Institute, University of Zurich, Switzerland

**Correspondence:**

Thomas F. Riegler

ZHAW Zurich University of Applied Sciences, School of Health Sciences, Institute of Physiotherapy

Katharina-Sulzer-Platz 9

8401 Winterthur

Switzerland

E-Mail: thomas.riegler@zhaw.ch

**Table S1: Examples of key quotes of each category and subcategory**

| Category | Representative quote |
| --- | --- |
| **Staying well with PF** |  |
| Understanding the disease | Words that people with PF understood, remembered, and use themselves to describe the disease: *“incurable”, “unknown origin (idiopathic)”, “scarring”, “rigid”, “alveoli harden”, “preventing effective gas exchange”, “lung looks like a spiderweb and gets compact”, “reduced lung capacity, which also means reduced breathing capacity”, “lung deterioration leads to low oxygen levels, affecting all organs”, “there may be progressive deterioration or exacerbations.”* (P1-11) |
| Life Expectancy | One participant about the question about life expectancy answered by a medical doctor: *“She said:’Yes, you will die from this fibrosis, that’s for sure.’ There’s nothing to discuss, right? So… she didn’t say when, but she basically wanted to tell us that people who have this… they are just condemned to death. Others too, right? But these especially.”* (P5)  About thoughts of the uncertain life expectancy*: “Of course, I want to live a long time. Not that I think I’ll die in a month or in a year. But even if... But of course I can’t really say when... But even if that were to happen, that means I would have to… I try to… I hope that it will not be so soon for me… sometimes I panic, when I think about that.”* (P2)  One participant about what information he most importantly needed when it comes to his disease*: “How much longer I can live, for example. …with these lungs. I am very interested in how it roughly happens and especially how the end unfolds – whether it’s suffocation or how it happens. I don’t know, I’ve never heard about it. That would certainly be useful. At least for me; whether how I use this information is my own concern.”* (P6)  *“I was in pulmonary rehabilitation, and I took this opportunity to learn even more about the disease. What seemed to interest me most was the question of dying. Because I thought to myself, if the lungs keep losing function, then one day I will die from shortness of breath, and that wasn’t a very comforting thought for me. […] I was told that with morphine, they have a method that they could use palliatively so that I wouldn’t feel the shortness of breath anymore before I died. Then I thought to myself, ‘okay, then that’s fine, then I don’t need to worry about it any further.’ ”*  (P1) |
| Attitude towards life | On participating in life and going out: *“Sure, I don’t go out much anymore, it’s a bit like that…you have to somewhat limit your life, right.”* (P4)  About symptoms limiting daily life and dealing with the disease: *“I take each day as it comes and try to stay calm. […]* The best thing is probably not to think too much about it.” (P7)  On dealing with the disease and intrusive thoughts: *“And always say to myself ‘stay positive’. I always say it, again and again, ‘always, stay positive’.”* (P3)  About information on travelling with PF: *”I want to know what is possible. In what condition that is possible. How long, and, and, and.“* (P11)  On adapting their approach and mindset to keep travelling: *“Before the diagnosis I used to go for a quick weekend to Paris,.. or to Hamburg, Berlin, or something like that. Nowadays it’s just the case that… I need more time. So, if I go on a city trip now, for example, I make sure that I can travel there without stress. Two, three, at least four to five days there, and then a stress-free return trip. This way I make sure I don’t put my body under stress. For example, running for the train at the last second or something like that – no way, that’s just not possible anymore. I’d rather wait ten minutes on the platform and take everything easy than rush in the last two minutes.”* (P9)  On lacking information on financial burdens due to the disease: *“Something that is missing is… the financial aspect and what the disease brings in that regards. Besides the health issues.”* (P8)  On having to fight for your needs being a patient: *“Yes, I really had to learn to fight for myself, to look out for myself,… so that I get the information that I need, right.”* (P8) |
| Medication and its side effects | On effects of medication: *“I feel like the side effects of the drugs are often more impactful than the intended effect itself...”* (P7)  About information on side effects of antifibrotic medication: *“I know that it’s supposed to help me, but the side effects aren’t really mentioned at all. They are listed, of course, but it doesn’t affect everyone the same. One person feels sick, another has other problems and can’t tolerate it. Something about the information should be improved here.”* (P5)  About participation being limited by side effects: *“And I was never sure whether I might… have an accident somewhere. When I took two loperamide, it went reasonably well. But I was never certain, when I went for a walk, whether it would really be safe or not. Also,.. I have lost my appetite, the desire to eat due to this medication.”* (P1)  On costs for antifibrotic medication: *“The thing about those pills; it’s insane how much they cost.”* (P10) |
| Nutrition | *“A new program about nutrition. That might not be bad. And not about nutrition in general terms or that pyramid thing. That’s old news. But in dialogue with all the medications together. That’s something I feel is missing.”* (P5)  “*I wouldn’t know anything about nutrition, and I never got any information about that. I try to eat less salt, and things like that.. but, I don’t know.”* (P10)  *“I received some list. But up until now, I’ve always eaten like this… well, properly, and normally. That works perfectly.”* (P6) |
| Peer support | *“I wouldn’t know what we would want to do there. Or, well, I would participate, but from my point of view it’s not really necessary. Such a group of affected people, or, self-help groups? I don’t know, what should I do there with people who have pulmonary fibrosis, celebrate my illness in a self-help group? And I actually don’t have much interest in that.”* (P1)  *“I think you’re a bit on your own when you have something like this. You read on the Internet, and basically—the Internet is on the one hand an amazing resource for everything you can find. But on the other hand, it naturally creates more doubts. Would this be something for me? Is this the right thing? Should I, should I not? So, I would say that exchanging with others would probably be very useful. It would probably also help me to deal with this situation better—yes, to get back to feeling whole again.”* (P9)  *“I’ve sometimes thought it would be good to have information somewhere… or a contact person who has already done or gone through it. Someone who is really an expert you can ask one-on-one.”* (P8) |
| **Keeping fit and strong with PF** | *“I was told to get as much exercise as possible… with the necessary rest breaks. All I remember is simply; exercise is good.”* (P6) |
| Barriers | *“You know, when you have so little breath, it’s just a vicious cycle…to still be able to keep yourself fit is very hard.”* (P8)  *“I don’t think I need to ride the bike for 20 minutes every day now. I don’t know what that would be good for.”* (P5)  *“I don’t know, often I don’t feel so well. Most often I do what’s necessary and then that’s it.”* (P2) |
| Facilitators | On what would help facilitate more physical activity: *“A brochure, or something that shows what one can do, or should do,… for specifically this situation with this disease.”* (P4)  On going out and being physically active: *“I visit my friends in the nearby area. I can still manage that, without getting too out of breath. But yes, it’s not possible every day.”* (P7) |
| **Using oxygen therapy** | A participant on what he understood why he needs supplemental oxygen: *“When the lungs no longer function... when the oxygen level is no longer right, it inevitably affects other organs too. Or the other organs then get weakened, like the kidneys, the heart, the whole system, you know. Because everything has to work together. They already explained that to me. That’s why I was switched to oxygen, so that I have a bit more oxygen in my blood. Because for quite a while, there just wasn’t enough anymore, you know.”* (P3) |
| Positive and negative impacts of oxygen therapy | An example for negative impacts of supplemental oxygen: *“When I go out, I have to take a backpack with me. That’s maybe more of a hindrance than I had imagined.”* (P5)  An example for positive effects of supplemental oxygen: *“I vacuum once a week, half of our apartment. And when I vacuum, I also use the backpack now, so that I can do it better without interruptions, when I use the oxygen.”* (P1) |
| Experimenting with oxygen dosage | *“I just tried not to take too much oxygen, so that the lungs still have to work at least somewhat on their own. But then, at [institution name 1], they told me that I really have to be careful.”* (P3)  *“Sometimes I try to go to bed without oxygen. If I can breathe well, that means I have no difficulties, then I try to sleep.”* (P2) |
| Traveling with supplemental oxygen | About the first time flying with supplemental oxygen: *“Until we were actually on the plane, I had it on, but then not anymore, because I thought, well, it’s fine I’m just sitting. But after we took off, maybe 20 minutes into the flight, I got a terrible headache. I measured my oxygen saturation, and it was at 80, so I realised I to use the oxygen.”* (P11)  On difficulties finding help in organising supplemental oxygen for a vacation: *“For example right now, next week we’re going up to [a city in the mountains], on a ski vacation, since there’s still snow. So it was about getting oxygen delivered there. First we asked the Lung Association, and they said it wasn’t their responsibility, I should sort it out with, [Person X] somehow. Before it used to be [Person Y], but not anymore. Now I should check with her, and then an email came saying they don’t deliver up there. And then I thought, well hello, what’s that supposed to mean? Before they said it was possible…”* (P8) |
| **Managing symptoms** |  |
| Breathlessness | A participant on the lack of self-management strategies for his breathlessness: *“I just take breaks; otherwise, I really haven’t figured out anything that helps my shortness of breath.”* (P7)  On positions that help alleviate breathlessness: *“So we got the x variants from [pulmonary rehabilitation] for relief or something. And I just took the one that fits me or makes sense for me.”* (P8)  A participant on needing more tips, techniques, or strategies to manage dyspnoea: *“How can I, for example, possibly prevent that? What do I need to consider more during the day, or what shouldn’t I do at all during the day, so that this breathlessness flare-up doesn’t happen? Or various hints and tips that could, for example, trigger shortness of breath. […] Like, when I have shortness of breath, what do I need to pay attention to, and how can I bring it back to a normal rhythm as quickly as possible?”* (P9) |
| Cough | A participant on feeling stigmatised due to her cough attacks: *“Then I cough when I get on the bus, and people have sometimes given me very angry looks. That’s a problem.”* (P11)  About fatiguing dry cough: “*The dry cough,… it’s so irritating and it makes me extremely tired.”* (P2)  A participant on having no chance to control his dry cough: *“I can only wait until the cough attack is over. Cough syrup and all those drugs never helped me at all.”* (P8)  On trying to suppress the cough: *“So I try to suppress it. Most of the time, that doesn’t work.”* (P9)  One participant on successfully reducing his cough with self-management: *“When I do my breathing and strength exercises at first I have to dry cough a lot, but afterwards it gets less and less until it stops.”* (P1) |
| Fatigue | On feeling fatigued and exhausted: *“The whole body just gets heavy. First the legs, and then the arms. And then the whole body. It’s like there’s lead hanging on you. And then you have to recover again before you can go again.”* (P3)  A participant on trying to use a type of pacing to manage his energy: *“I help my wife with housework. I get tired, but with that I take my time – I do the movements slowly, so that I can do more. Of course, at some point I get tired.”* (P2)  On trying to use a type of energy conservation technique: *“I don’t really have a strategy. I just take each day as it comes and just try to use as little energy as possible, so that some would remain for later in the day. Sometimes it works better, sometimes less. But basically, I don’t have a strategy for that.”* (P8)  About actively trying to be more physically active to reduce fatigue: *“More activity, make the system maybe work a little, use some energy, and generally live a bit more, and put a bit of strain on the lungs, you know. If I just sit around on the chair, or lie on the bed, then it’s not getting better either.”* (P10) |
| Symptoms of anxiety, depression, and panic | *“So, sometimes it hits you hard. Yes, sometimes you just think, what’s the point of all this. And somehow you still want to do it, you want to keep living a little, and then you end up somewhere in between.”* (P8)  About sharing your thoughts and emotions with healthcare professionals: *“That often helps, to simply share your thoughts and feelings with healthcare professionals and not just sit there and do nothing about it or against it.”* (P4)  On reframing uncertainties about illness and fate: *“I’ve actually never been depressed and I have no reason to become depressed because of this illness. For me, these are simply… these are life questions, you know.”* (P1)  One participant on avoiding the topic of PF: *“I don’t really want to know more about it. It pulls you into a hole.”* (P10) |
| **Sources of information** | On getting primary information and future contact from a healthcare professional: *“But she told me, if I need help, she is always available. She left her phone number. And I have to say, I think that’s very, very good.”* (P2)  A participant describing the sense that no one can offer useful information: *“I think, they don’t really know either. The healthcare professionals are at the limit of their knowledge.”* (P7)  On finding anxiety-inducing information on PF on the internet: “I myself didn’t google. That was my daughters who did that. Only later did I do it. They knew more than I did. And it also makes the relatives a little scared, you see?” (P6)  On not trusting the information on the internet*: “Or from the Internet, you can take X things, and there’s a lot out there… but I don’t know if it is reliable information,… it’s a very difficult thing.”* (P8)  One participant on getting too much and too distributed information: *“She gave me brochures and stuff, basically a full library.”* (P10) |
| **Modes of information delivery** | A participant on a blended PESM solution: *“I would find it absolutely useful. Because you can look up what interests you most at the moment, and discuss it at the next appointment.”* (P8)  A participant on reliable digital information resources on PESM for PF: *“I think that would be a very good thing, and I would participate in such a programme.”* (P2)  About having visual resources for techniques to refer to: *“Maybe visually so that you can perhaps refer back to this video if you suddenly feel unsure; ‘Am I doing it right now? Do I have to do it two or three times?”* (P9) |
